# Prolonged leg bending impairs endothelial function in the popliteal artery

**DOI:** 10.14814/phy2.13478

**Published:** 2017-10-23

**Authors:** Lauren K. Walsh, Robert M. Restaino, Luis A. Martinez‐Lemus, Jaume Padilla

**Affiliations:** ^1^ Nutrition and Exercise Physiology University of Missouri Columbia Missouri; ^2^ Medical Pharmacology and Physiology University of Missouri Columbia Missouri; ^3^ Dalton Cardiovascular Research Center University of Missouri Columbia Missouri; ^4^ Child Health University of Missouri Columbia Missouri

**Keywords:** Blood flow, endothelial dysfunction, shear stress, sitting vasculopathy

## Abstract

Uninterrupted sitting blunts vascular endothelial function in the lower extremities; however, the factors contributing to this impairment remain largely unknown. Herein, we tested the hypothesis that prolonged flexion of the hip and knee joints, as it occurs during sitting, and associated low shear stress and disturbed (i.e., turbulent) blood flow caused by arterial bending, impairs endothelial function at the popliteal artery. Bilateral measurements of popliteal artery flow‐mediated dilation (FMD) were performed in 12 healthy subjects before and after a 3‐h lying‐down period during which one leg was bent (i.e., 90‐degree angles at the hip and knee) and the contralateral leg remained straight, serving as internal control. During the 3‐h lying down period, the bent leg displayed a profound and sustained reduction in popliteal artery blood flow and mean shear rate; whereas a slight but steady decline that only became significant at 3 h was noted in the straight leg. Notably, 3 h of lying down markedly impaired popliteal artery FMD in the bent leg (pre: 6.3 ± 1.2% vs. post: 2.8 ± 0.91%; *P* < 0.01) but not in the straight leg (pre: 5.6 ± 1.1% vs. post: 7.1 ± 1.2%; *P* = 0.24). Collectively, this study provides evidence that prolonged bending of the leg causes endothelial dysfunction in the popliteal artery. This effect is likely secondary to vascular exposure to low and disturbed blood flow resulting from arterial angulation. We conclude that spending excessive time with legs bent and immobile, irrespective of whether this is in the setting of sitting or lying‐down, may be disadvantageous for leg vascular health.

## Introduction

Recent evidence indicates that prolonged sitting (i.e., three hours or more) impairs endothelial function in the popliteal artery (Restaino et al. 2015, 2016; Morishima et al. 2016, 2017), an artery that is highly susceptible to the development of atherosclerosis (Ross et al. 1984; Stary et al. 1995; Moore 2002; Aboyans et al. 2011). Because endothelial dysfunction is a key element in the initiation and progression of peripheral artery disease (McLenachan et al. 1991; Widlansky et al. 2003), a better understanding of the mechanisms and factors contributing to leg endothelial dysfunction with excessive sitting is of paramount significance.

A consistent finding is that leg blood flow is prominently reduced during sitting (Restaino et al. 2015, 2016; Morishima et al. 2016, 2017). Given that shear stress is a pivotal signal for maintaining endothelial health (Cheng et al. 2004, 2006; Laughlin et al. 2004, 2008; McAllister et al. 2005; Woodman et al. 2005; Chatzizisis et al. 2007; Siasos et al. 2007; Ridger et al. 2008; Davies 2009; Tinken et al. 2010; Newcomer et al. 2011; Jenkins et al. 2012; Green et al. 2017), it is reasonable to propose that reduced leg blood flow and shear stress with sitting contributes to endothelial dysfunction in the lower extremities. Indeed, we recently showed that preventing the reduction in leg blood flow during prolonged sitting, with local heating of the foot (Restaino et al. 2016) or fidgeting (Morishima et al. 2016), abolished the impairment in endothelial function at the popliteal artery. Despite the recognition that sitting‐induced leg endothelial dysfunction is in part attributable to the reduction in leg blood flow and shear stress, the factors contributing to the increased lower‐limb vascular resistance during sitting remain largely unknown. Lack of leg muscle contraction for extended periods undoubtedly contributes to the steady decay in limb blood flow during sitting. However, the observation that popliteal artery blood flow is immediately reduced when transitioning from the supine to the sitting posture (Vranish et al. 2017), and largely reestablished upon return to the supine position after prolonged sitting (Restaino et al. 2015, 2016; Morishima et al. 2016, 2017), suggests that biomechanical factors may also be implicated in the decline of popliteal artery blood flow during sitting. As such, we postulate that flexion of the hips and knees with sitting, and associated arterial bending, may obstruct limb blood flow (Morishima et al. 2017). Arterial bending not only causes a reduction in limb blood flow but, presumably, it also creates a region of flow disturbance (i.e., turbulence), immediately distal to the site of bending. Notably, it is well‐established that turbulent blood flow, arising in geometrically irregular arterial regions such as branches, bifurcations and sharp curvatures, is atherogenic (Caro et al. 1969; Chatzizisis et al. 2007; Padilla et al. 2014). However, it is unknown whether prolonged bending of the hip and knee joints, as it occurs during extended periods of sitting, and consequent local hemodynamic changes, impairs endothelial function in the popliteal artery.

To address this question, herein, bilateral measurements of popliteal artery flow‐mediated dilation (FMD) were performed to determine endothelial function before and after a 3‐h lying‐down period during which one leg was bent (i.e., 90‐degree angles at the hip and knee) and the contralateral leg remained straight, serving as internal control. The subject was placed in the lying‐down position with the attempt to exclusively examine the effects of arterial bending, independent of gravitational forces naturally occurring with upright sitting. We hypothesized that endothelial dysfunction would be manifested in the popliteal artery of the bent, but not straight, leg. We reasoned that impaired popliteal artery endothelial function in the bent leg would be secondary to the hemodynamic changes subjected to that leg.

## Materials and Methods

Twelve young healthy subjects (men *n* = 8, women *n* = 4) recruited from the University of Missouri campus participated in this study (Age: 26.1 ± 1.1 years; Height: 172.4 ± 2.2 cm; Weight: 73.6 ± 1.9 kg; BMI: 24.6 ± 0.4 kg/m^2^). All experimental procedures and measurements conformed to the Declaration of Helsinki and were approved by the University of Missouri Health Sciences Institutional Review Board. Prior to participating in the study, each subject provided written informed consent. Subjects were recreationally active, nonsmokers, with no history or symptoms of cardiovascular, pulmonary, metabolic, or neurological disease as determined from a medical health history questionnaire. No subjects reported taking medications.

### Experimental procedures

**Figure 1:**
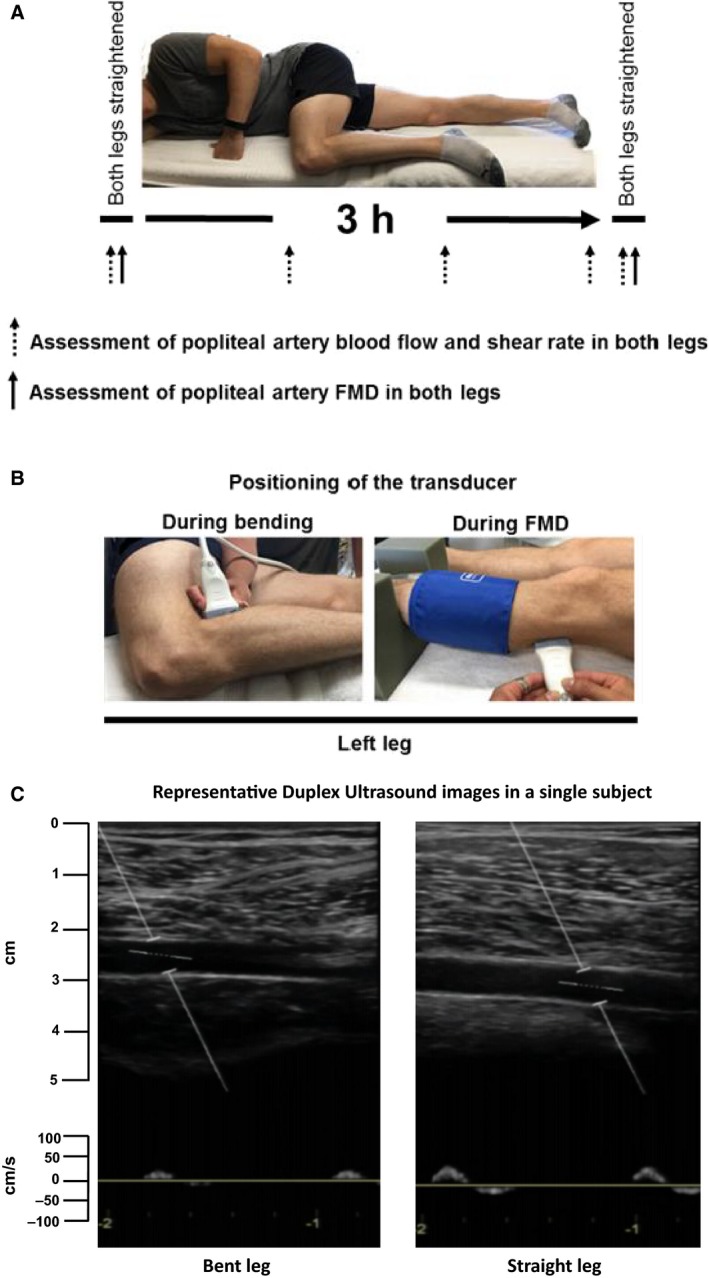
Experimental design. (A) Popliteal artery blood flow, shear rate, and flow‐mediated dilation (FMD) were assessed in both legs at baseline and immediately after a 3‐h experimental period of lying down with one leg bent at a 90‐degree angle at the hip and knee while the contralateral leg remained straight, as illustrated in the picture. Baseline and post measures were performed in the supine position with both legs straightened. The post measurements were initiated immediately (within 5 min) after reestablishing the supine position and the order of measures was randomized between legs within each subject. During the 3‐h experimental period, popliteal artery blood flow and shear rate were assessed in both the straight and bent legs at 1, 2, and 3 h, as denoted in the schematic. (B) Positioning of the Doppler ultrasound transducer during measurements with the leg bent and also during FMD. FMD, flow‐mediated dilation. (C) Representative Duplex Ultrasound images of the popliteal arteries from both bent and straight legs in a single subject at the two‐hour time point during the experimental intervention. For the bent leg, the section of the artery that is imaged is immediately distal to the site of bending (which occurs approximately 2–3 cm to the left of the sample volume).

A schematic of the study design is presented in Figure 1A, illustrating the positioning of the subject and sequence of measurements throughout the experimental visit. All women were tested on days 1–7 of their menstrual cycle following previously reported guidelines of FMD (Thijssen et al. 2011). Subjects were instructed to eat a light meal 2 or more hours prior to arriving to the laboratory. In addition, subjects were asked to refrain from caffeine and alcohol for at least 12 h, as well as from exercise for 24 h prior to the study visit. All study visits were performed in a temperature‐controlled room kept at 22–23°C. Upon arrival to the laboratory, subjects were placed in a supine position. After 20 min of resting quietly, popliteal artery diameter and blood velocity were measured using duplex‐Doppler ultrasound (Logiq P5; GE Medical Systems, Milwaukee, WI). An 11 MHz linear array transducer was placed over the popliteal artery just distal to the popliteal fossa (Fig. 1B). Simultaneous diameter and velocity signals were obtained in duplex mode at a pulsed frequency of 5 MHz and corrected with an insonation angle of 60 degrees. Popliteal artery FMD was assessed in both legs as previously described (Restaino et al. 2015, 2016; Morishima et al. 2016, 2017). Briefly, a rapid inflating cuff was placed on the lower leg (midpoint between knee and ankle). Two minutes of baseline hemodynamics were recorded and then the cuff was inflated to a pressure of 220 mmHg for 5 min. Continuous diameter and blood velocity measures were recorded for 3 min following cuff deflation. Recordings of all vascular variables were analyzed offline using specialized edge‐detection software (Cardiovascular Suite, Quipu srl., Pisa, Italy).

Following baseline FMD measurements, subjects were maintained in the lying down position with one leg bent at a 90‐degree angle at the hip and knee, while the contralateral leg remained straight, for 3 h, as illustrated in Figure 1A. Throughout the entire experimental period, a study representative monitored the subject to ensure no leg movements or muscle contractions occurred in either leg. Right and left legs were randomly assigned to the condition of bent or straight leg. Throughout the course of the study period, popliteal artery diameter and blood velocity were measured on both legs every hour in order to quantify popliteal artery blood flow and shear rate. The position of the legs remained in their experimental conditions during blood flow measurements, as clear images were able to be captured just distal to the bent knee as well as in the straight leg, while they remained in position (Fig. 1C).

Following the 3‐h experimental period, subjects were repositioned into the supine position and straightened their bent leg. FMD measurements were then immediately (within 5 min) repeated. The order of FMD assessments was randomized between straight and bent legs within each subject. Once FMD was completed in one leg, the other leg was immediately set up for measurement (<5 min). The use of within‐subjects/between‐limbs design, previously employed by others (Thijssen et al. 2009a), provides greater experimental control of confounding factors thus reducing variability.

### Data analysis

Blood flow was calculated from continuous diameter and mean blood velocity recordings at each of the time points using the following equation: 3.14*(diameter/2)^2^ * mean blood velocity *60. Popliteal artery FMD percent change was calculated using the following equation: %FMD = (peak diameter‐base diameter)/(base diameter) *100. Shear rate, an estimate of shear stress without blood viscosity, was calculated as 4* mean blood velocity/diameter. For calculations of antegrade and retrograde shear rate, antegrade and retrograde time‐averaged blood velocities were used, respectively. Hyperemic shear rate area under the curve (AUC) up to peak diameter was calculated as a stimulus for FMD, as described (Pyke and Tschakovsky 2007).

### Statistical analysis

A two‐way (time x leg condition) repeated measures analysis of variance (ANOVA) with Tukey posthoc testing was performed on all dependent variables. FMD was also adjusted for hyperemic shear rate AUC via analysis of covariance (ANCOVA) in order to statistically control for the influence of shear stimulus on the FMD response (Atkinson et al. 2009). ANCOVA and ANOVA tests were performed using SPSS software (version 23). Significance was accepted at *P* ≤ 0.05. Data are expressed as means ± SE.

## Results

**Figure 2:**
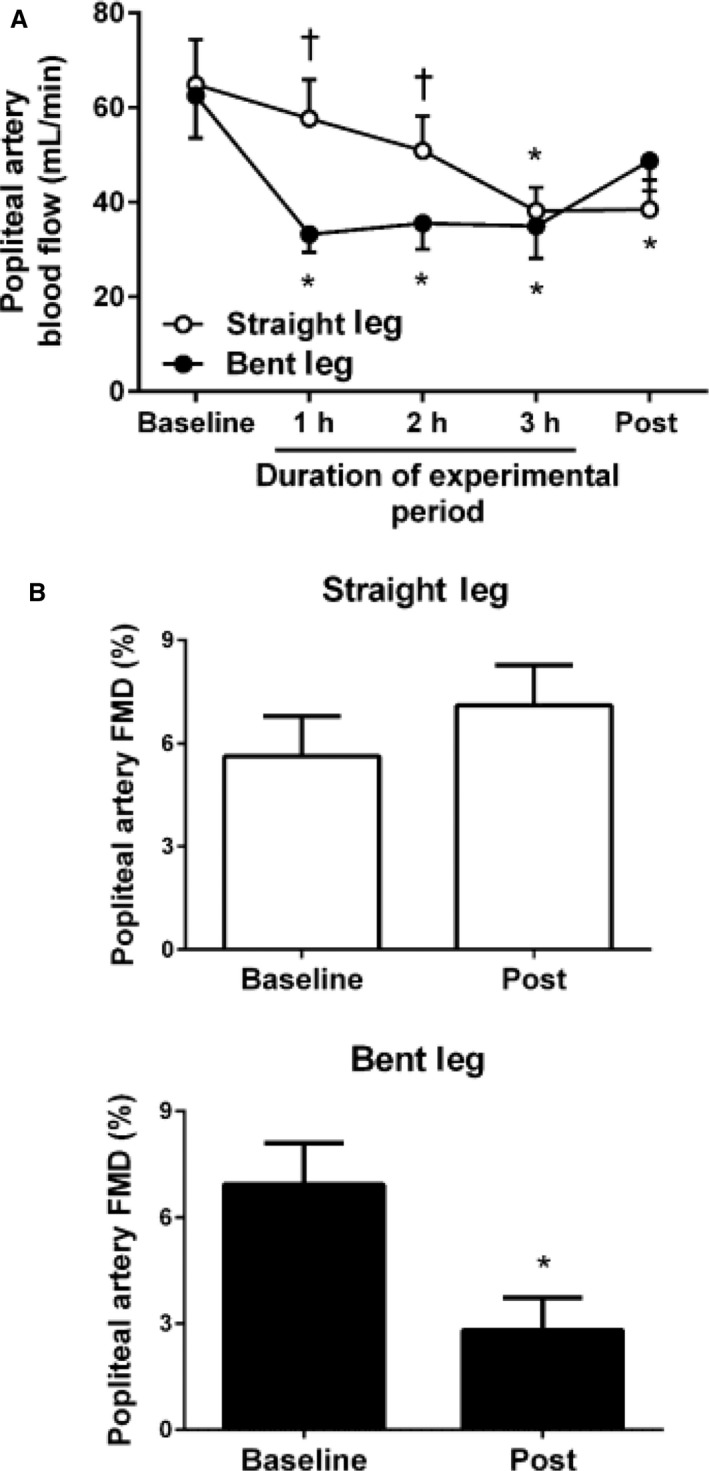
Popliteal artery blood flow and FMD in the straight and bent legs. (A) Popliteal artery blood flow at baseline, during the experimental period, and immediately thereafter in the straight and bent legs. Baseline and post measures were performed in the supine position with both legs straightened. (B) Popliteal artery FMD in the straight leg and bent leg at baseline and immediately after the 3‐h experimental period (post). Data are expressed as means ± SE. *P < 0.05 versus Baseline, ^†^ *P* < 0.05 between legs.

Over the course of the 3‐h experimental period, popliteal artery blood flow (Fig. 2A) and mean shear rate (Table 1) were markedly reduced in the bent leg (*P* < 0.05). Upon straightening of the bent leg after the 3‐h bending period, blood flow and shear rate levels were partially restored towards baseline values (Fig. 2A and Table 1). In contrast, in the straight leg, popliteal artery blood flow and shear rate slightly but progressively declined over time with a significant decrease noted in both variables at hour 3 (Fig. 2A and Table 1, *P* ****<** **0.05).

**Table 1:** Popliteal artery hemodynamics at baseline, during, and after 3 h of intervention

		Baseline	Duration of experimental period			Post	ANOVA
			1 h	2 h	3 h		
Mean shear rate (sec^−1^)	Straight leg	57.96 ± 5.6	51.41 ± 6.3†	47.31 ± 6.3†	38.24 ± 4.3*	39.1 ± 5.9*	Time: *P *=* *0.005 Leg: *P *=* *0.05 Interaction: *P *=* *0.09
	Bent leg	53.52 ± 3.2	35.44 ± 4.4*	36.42 ± 4.3*	33.97 ± 3.3*	43.7 ± 3.5*	
Antegrade shear rate (sec^−1^)	Straight leg	85.2 ± 8.6	74.4 ± 6.9†	76.9 ± 7.6†	68.5 ± 6.5*	63.7 ± 9.7*	Time: *P *=* *0.001 Leg: *P *=* *0.14 Interaction: *P *=* *0.05
	Bent leg	82.13 ± 5.8	50.25 ± 4.5*	53.4 ± 5.8*	55.8 ± 7.7*	68.4 ± 8.3*	
Retrograde shear rate (sec^−1^)	Straight leg	−33.3 ± 9.3	−31.2 ± 7.9†	−30.4 ± 6.3†	−30.4 ± 6.2	−27.1 ± 6.7	Time: *P *=* *0.42 Leg: *P *=* *0.16 Interaction: *P *=* *0.32
	Bent leg	−28.6 ± 6.1	−14.8 ± 4.8*	−16.9 ± 3.8*	−21.9 ± 6.1*	−24.2 ± 6.1	
Basal diameter (cm)	Straight leg	0.56 ± 0.03	0.55 ± 0.03	0.57 ± 0.03	0.54 ± 0.03	0.58 ± 0.03	Time: *P *=* *0.98 Leg: *P *=* *0.76 Interaction: *P *=* *0.94
	Bent leg	0.55 ± 0.02	0.57 ± 0.03	0.57 ± 0.03	0.57 ± 0.02	0.56 ± 0.03	
Hyperemic shear rate AUC (A.U.)	Straight leg	39871 ± 12326				34912 ± 6985	Time: *P *=* *0.29 Leg: *P *=* *0.41 Interaction: *P *=* *0.91
	Bent leg	33619 ± 5935				34206 ± 7064	
ANCOVA‐ corrected FMD (%)	Straight leg	5.72 ± 1.09				7.11 ± 1.09	Time: *P *=* *0.22 Leg: *P *=* *0.18 Interaction: *P *=* *0.01
	Bent leg	6.89 ± 1.09				2.83 ± 1.09*	
Heart rate (BPM)		60 ± 2	55 ± 2	59 ± 2	58 ± 3	54 ± 3	Time: *P* = 0.74

A.U., arbitrary units.

Values are means ± SE. ANCOVA‐ corrected flow‐mediated dilation (FMD) data are adjusted for hyperemic shear rate area under the curve (AUC). *P < 0.05 versus Baseline (0 h), †*P* < 0.05 versus straight versus bent leg.

Importantly, as illustrated in Figure 2B, maintenance of the 90‐degree angle at the hip and knee for 3 h caused a significant impairment in popliteal artery FMD in that bent leg (*P* < 0.01 vs. baseline) but not in the contralateral leg kept straight (*P* = 0.24 vs. baseline; *P* = 0.01 for time x leg interaction). Hyperemic shear rate AUC was similar between legs before the 3‐h experimental period and remained unchanged thereafter (Table 1). FMD corrected for hyperemic shear rate AUC by ANCOVA did not affect the interpretation of the findings (Table 1). No significant changes were observed in popliteal artery diameter over time and no differences between legs were detected across time points (Table 1). Heart rate was not significantly affected throughout the experimental protocol (Table 1).

## Discussion

This study provides evidence that prolonged (3 h) bending of the hip and knee joints, as it occurs with sitting, is sufficient to impair endothelial function in the popliteal artery. Popliteal artery endothelial dysfunction with bending of the leg is likely attributed to low shear stress and blood flow disturbance caused by the arterial angulation.

It is becoming well‐established that uninterrupted sitting impairs endothelial function in the lower extremities (Restaino et al. 2015, 2016; Morishima et al. 2016, 2017). While the factors contributing to leg endothelial dysfunction with lengthy sitting are not fully elucidated, current data suggest that reduced limb blood flow and shear stress during sitting is a primary factor underlying leg vascular dysfunction (Morishima et al. 2016; Restaino et al. 2016). A conceivable explanation for the reduction in leg blood flow during sitting is the lack of skeletal muscle activity in the lower extremities and ensuing reduction in energy demand. Consistent with this notion, herein, we showed that lying down without leg movement also leads to a steady decay in leg blood flow over the 3‐h period (Fig. 2A). This progressive reduction in leg blood flow was likely attributed to the complete muscular inactivity of the limb during the 3 h. However, the decline in leg blood flow with sitting over the same time frame (Restaino et al. 2015; Morishima et al. 2016, 2017; Vranish et al. 2017) is much more pronounced to that during lying down, suggesting that other factors beyond the fall in energy demand are implicated. Indeed, data from this study reveal that an important determinant of decreased leg blood flow during sitting is the flexion of the hips and knees and associated arterial bending. That is, here we showed that recapitulation of the sitting position (i.e., hip and knee joints bent to 90 degrees) causes a marked reduction in popliteal artery blood flow. Of note, this reduction in blood flow in the bent leg occurred with the subject lying down, suggesting that arterial bending *per se*, independent of gravitational forces inherent to upright sitting, is sufficient to limit limb blood perfusion. Notably, maintenance of leg bending for 3 h impaired endothelial function in the popliteal artery, an effect that was not observed in the contralateral leg kept straight (Fig. 2B).

As abovementioned, it is worth noting that the leg maintained straight also exhibited a progressive decline in blood flow over time, likely attributed to the sustained inactivity of the lower extremities. Of importance, this magnitude of reduced leg blood flow and shear rate in the straight leg was insufficient to impair popliteal artery endothelial function, although after 3 h of lying down, both legs displayed similar levels of attenuated blood flow. This observation could be interpreted as evidence that the impairment in endothelial function at the popliteal artery with leg bending results from the accumulated reduction in mean shear stress over the previous 3 h rather than from the more immediate levels of shear prior to post FMD measures. Alternatively, it could be interpreted that the impairment with leg bending was not solely caused by the reduction in blood flow and shear stress. That is, it is plausible that popliteal artery endothelial dysfunction with prolonged leg bending was in part mediated by the disturbance of flow (i.e., increased turbulence) occurring immediately distal to the site of arterial bending; that is,, the site of FMD measurement. In this regard, we know from post‐mortem human studies that the most atheroprone segments of the vasculature are regions characterized by presence of flow turbulence, including branch points, bifurcations, and sharp curvatures (Caro et al. 1969; Chatzizisis et al. 2007; Padilla et al. 2014). Studies in animal models also demonstrate that subjecting the vasculature to low shear stress and disturbed blood flow, by application of a partial occluder, increases vulnerability to atherosclerotic lesions (Cheng et al. 2006). Taken together, it is possible that constant bending of the leg perturbs endothelial function at the popliteal artery as a result of vascular exposure to reduced mean shear stress and increased turbulent blood flow.

Peripheral arteries exhibit a triphasic flow pattern primarily characterized by a large antegrade flow during systole, followed by a brief episode of retrograde flow in early diastole and a subsequent phase of antegrade flow in mid‐ and late diastole that is much smaller in magnitude (Mc 1955; Blackshear et al. 1979). Over the last decade, we (Padilla et al. 2010, 2011; Young et al. 2010; Simmons et al. 2011; Casey et al. 2012; Jenkins et al. 2013) and others (Green et al. 2005; Thijssen et al. 2009a, 2009b, 2014, 2015; Tinken et al. 2009; Scholten et al. 2014; Schreuder et al. 2015) have identified physiological factors that influence the shape of the flow wave as well as examined its impact on vascular function. Previous work indicates that increased retrograde shear stress, typically associated with a reduction in antegrade and mean shear, impairs endothelial function (Thijssen et al. 2009a, 2015). Thus, given the evidence that the pattern of flow in peripheral conduit arteries is an important determinant of vascular health, we examined if leg bending resulted in alterations in the magnitude of popliteal artery antegrade and retrograde shear rate assessed immediately distal to the site of bending. As displayed in Table 1 and Figure 1C, bending resulted in a pronounced reduction in antegrade shear, contributing to the reduction in mean shear. The reduction in antegrade shear in the bent leg was also accompanied by a reduction in retrograde shear. Therefore, impaired vascular function downstream from the site of arterial angulation cannot be attributed to an increase in retrograde shear stress. Rather, as discussed above, the impairment in vascular function is likely attributed to a combination of low time‐averaged shear stress and increased turbulent flow.

Findings from the present investigation have noteworthy clinical implications. First, this work supports arterial bending as a potential causative factor to sitting‐induced leg endothelial dysfunction. Second, these findings stimulate the novel idea that body position during sleeping hours may have ramifications for leg vascular health. Indeed, on the basis of the present data, it is reasonable to hypothesize that sleeping with legs bent is a predisposing factor to leg vascular disease, which may manifest when additional systemic risk factors (e.g., aging, type 2 diabetes, smoking) are superimposed. However, this is an unexplored area of study and thus large cohort studies and laboratory‐based research are needed to examine this interesting hypothesis. Future studies should also examine whether sitting with the legs unbent (i.e., “sitting with the legs up”) would be a more vascular‐protective form of sitting than with legs bent. In addition, further studies are needed to evaluate the influence of age and sex hormones in modulating vascular responses to leg bending.

Some considerations related to the experimental design should be highlighted. While experiments were performed with the subject placed in the lying‐down position to examine the influence of leg bending with minimal influence of gravitational forces (Fig. 1A), we should acknowledge that the position of the bent leg over the straight leg may have resulted in slight differences in hydrostatic pressure between legs, thus contributing to some of the differences in blood flow. The change in positions throughout the experimental protocol (e.g., from lying down sideways to lying down supine) might have also contributed to some of the changes in leg blood flow. We decided to perform the measurements of FMD with the subject positioned supine to adhere to current FMD guidelines and to be consistent with previous studies from our group and others. Future studies should aim to quantify the changes in turbulent blood flow using other in vivo imaging techniques.

In conclusion, this study provides the first evidence that prolonged bending of the leg causes endothelial dysfunction in the popliteal artery. This effect is likely due to vascular exposure to low and disturbed blood flow resulting from arterial angulation. Together, these findings support the hypothesis that the previously reported sitting vasculopathy in the popliteal artery (Restaino et al. 2015, 2016; Morishima et al. 2016, 2017) may be attributable to limb bending. Accordingly, for preservation of leg vascular health it appears that people should limit extended periods of time with legs bent and immobile, regardless of whether this is in the setting of sitting or lying‐down.

## Conflicts of Interest

None.
